# Measurement and analysis of partial lightning currents in a head phantom

**DOI:** 10.1371/journal.pone.0223133

**Published:** 2019-09-26

**Authors:** René Machts, Alexander Hunold, Christian Drebenstedt, Michael Rock, Carsten Leu, Jens Haueisen

**Affiliations:** 1 Institute of Biomedical Engineering and Informatics, Technische Universitaet Ilmenau, Ilmenau, Germany; 2 Group for Lightning and Overvoltage Protection, Technische Universitaet Ilmenau, Ilmenau, Germany; 3 Research Unit High-Voltage Technologies, Technische Universitaet Ilmenau, Ilmenau, Germany; Arizona State University, UNITED STATES

## Abstract

Direct lightning strikes to the human head can lead to various effects, ranging from burnings to death. The biological and physical mechanisms of a direct lightning strike in the human head are not well understood. The aim of this paper is to design an experimental setup to measure the spatial and temporal current distribution during a direct lightning strike to physical head phantoms to establish normative values for personal lightning protection equipment design and testing. We created head phantoms made of agarose, replicating the geometric and dielectric properties of scalp, skull, and intracranial volume. The bases of the three compartments were galvanically contacted via copper electrodes to measure the current per compartment. We used pulse generators to apply aperiodic voltage and current signals that modelled lightning components. Our experiments indicated that the scalp compartment was exposed to the current with a fraction of 80–90%. The brain and skull compartments were exposed between 6–13% and 3–6% of the total measured current respectively. In case of a flashover, most of the current (98–99%) flowed through the discharge channel. Unlike previous theoretical estimates and measurements in technical setups, we observed considerably longer times for the flashover to build up. In our experiments, the time to build up a fully formed flashover varied from approximately 30–700 μs. The observed current patterns in cases without and with flashover provided information on regions of possible damage in the human head. Consequently, we identified the phenomenon of a flashover as a potential mechanism for humans to survive a lightning strike. Our measured current distributions and amplitudes formed the base for normative values, which can be used in later experimental investigations regarding the possibilities of individual lightning protection equipment for humans.

## Introduction

The chance of being struck by lightning increases when humans are outdoors in a thunderstorm. They are especially exposed to lightning strikes directly in the head in open elevated areas [1–3]. A direct lightning strike can cause physical damages like burns on the skin, fractures in the skull, or haemorrhage in the brain due to the heat and explosive effects, as described in several pathological diagnosis reports and other literature [4–10]. These effects are caused by currents up to 200 kA and voltages of 1 MV and higher in discharges of a few milliseconds [11–13].

Overall, the mortality of lightning injury is only about 10% [14,15] but increases to about 30% when people are struck by lightning outdoors [1,2]. There are five known mechanisms of lightning injury: direct strikes accounting for 3–5% of the cases, side flashes for 30–35%, contact injury 3–5%, upward streamer 10–15% and ground current 50–55% [3,15]. A possible mechanism responsible for the relatively large chances of surviving a lightning strike despite the mentioned medical issues could be the formation of a flashover outside the body. In a flashover, most of the current flows outside the body and only a few Amperes flow inside the body [12,13], as estimated in a theoretical study by Uman [12]. This reduced current inside the body during a flashover might be small enough to not be lethal. This might be especially true for the head owing to the assumed additional protective effect of the skull caused by its low conductivity. The head is one of the most important parts of the human body and is most exposed during a direct strike or side flash. However, there are no reports in the literature on measured current distributions inside the head before and during a flashover in a direct lightning strike. Consequently, we aim to establish an experimental setup to measure current distributions for the main compartments of the human head using a physical head phantom. With these measurements, we want to establish normative values that can serve as a basis for later experimental investigations on the possibilities of lightning protection equipment for humans according to the standard DIN EN 1149 [16].

To achieve this aim, we have designed human head phantoms comprising scalp, skull, and intracranial compartments, mimicking the geometry, relative permittivity, and electric conductivity of real biological human tissue. In contrary to the homogeneous models used previously to investigate the flashover mechanism [17] the nonhomogeneous head phantoms allow to quantify the current distribution in the three compartments. Moreover, the head phantoms allow a qualitative analyses of visible damages per compartment. Consequently, we will be able to experimentally quantify the additional protective effect of the skull caused by its low conductivity. In addition to the currents flowing in the three head compartments, we measured the flashover current with an electrode outside and underneath the head phantom. With these measurements, we strive to obtain an experimental validation of the theoretical values proposed by Uman [12].

## Materials and methods

### Overview

**Fig 1:**
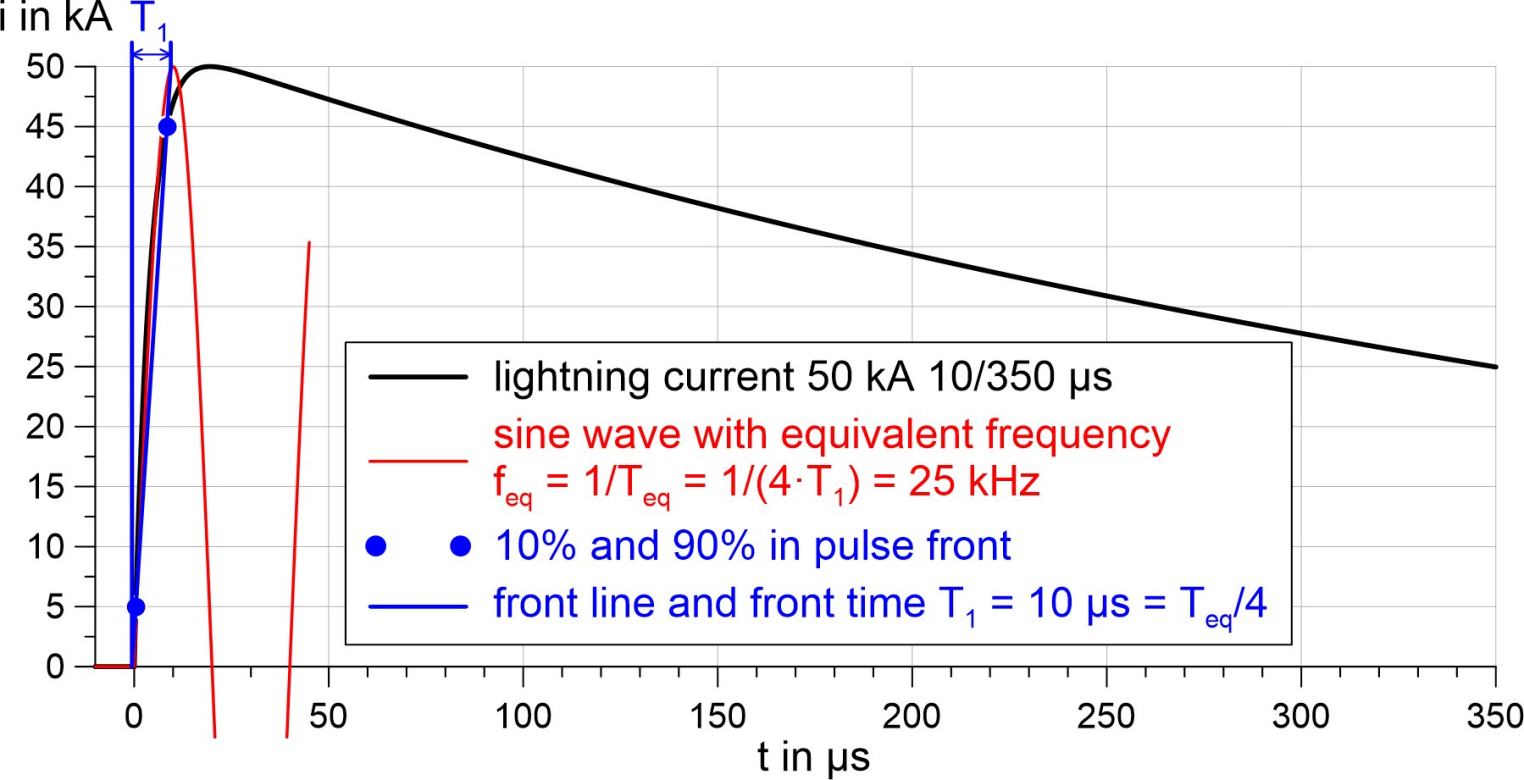
Calculation of the equivalent front frequency of a lightning pulse current. The front time (T_1_) of the 10/350 *μ*s lightning current is 10 *μ*s. The equivalent frequency (f_eq_) is based on a sine wave and the corresponding equivalent period (T_eq_).

We applied pulses to the head phantoms using three different pulse generators with different properties. A Marx generator delivered up to 450 kV and relatively low currents of up to 2 kA with an 1.2/50 μs voltage pulse; a combination wave generator delivered up to 12 kV and 6 kA with low noise (due to the compact design) and 1.2/50 μs voltage pulse or 8/20 μs current pulse; a 10/350 μs current pulse generator delivered up to 12 kV and 42 kA. The various generators emulated different aspects of a lightning flash. For example, the standardized positive first lightning current is defined by front time 10 μs, the maximum amplitude and time to half-value on tail 350 μs, identified as 10/350 μs according to the standard IEC 62305 [18]. The equivalent frequency is 25 kHz for the front of this pulse. Fig 1 illustrates the calculation of the equivalent frequency. After the peak, the frequency content decreases to 1 kHz and lower [11].

### Creation of head phantom

We created three-compartment head phantoms based on the computer tomography data of an individual human head similar to Tidswell et al. [19] and Sperandio et al. [20]. We segmented the inner and outer boundaries of the skull and the outer skin boundary from the CT data [21] using Seg3D (SCI Institute, Salt Lake City, UT, United States) and designed forms resampling these boundaries as negative moulds for the head phantom compartments in SolidWorks® (Dassault Systèmes, Vélizy-Villacoublay, France). We produced the moulds of the compartments through a fused deposition modelling process. The moulds are made of the polymer Lignin (GreenTec, Extrudr®, Lauterach, Austria). The casting process of the compartments started with the innermost compartment, the intracranial volume (brain), continued to the central compartment, the neurocranium (skull), and ended with the outermost compartment the scalp; in each case, the preceding compartment provided the base for the next compartment. These head phantoms resampled the geometry of the head and the dielectric properties of biological tissue. We used 2% agarose (Agarose Broad Range, Carl Roth GmbH + Co. KG, Karlsruhe, Germany) in deionized water as the basic phantom material. The dielectric properties of the compartments were set by doping the basic phantom material with different fractions of sodium chloride (NaCl), graphite (Graphite d50, Algin-Chemie e. K., Neustadt-Glewe, Germany), or carbon black (carbon fibre powder, EMV Vega, Recklinghausen, Germany). We used the mixtures (shown in Table 1) for creating head phantoms, in accordance to literature values for the dielectric properties. The dielectric properties were analysed with a Hewlett Packard 4284A 20 Hz to 1 MHz precision LCR meter (HP Inc., Palo Alto, CA, United States) in a two-plate method.

**Table 1:** Dielectric properties of the compartments of the two types of head phantoms in the range of 20 Hz to 1 MHz.

Phantom compartment	Mixture	Electric conductivity in S/m 20 Hz–1 MHz	Relative permittivity 20 Hz–1 MHz
**Head phantom Type I**			
**Brain Type I**	2 % agarose, 0.17 % NaCl	0.12–0.38	72.5·10^6^–930
**Skull Type I**	2 % agarose, 0.01 % NaCl, 4 % graphite	0.024–0.063	9·10^6^–196
**Scalp Type I**	2 % agarose, 0.17 % NaCl, 4 % carbon black	0.054–0.63	43·10^6^–1290
**Head phantom Type II**			
**Brain Type II**	2 % agarose, 0.17 % NaCl	0.12–0.38	72.5·10^6^–930
**Skull Type II**	2 % agarose, 0.01 % NaCl	0.015–0.048	8.1·10^6^–155
**Scalp Type II**	2 % agarose, 0.17 % NaCl	0.12–0.38	72.5·10^6^–930

Two types of phantoms were created, with (Type I) and without carbon additives (Type II). Type I phantoms had the advantage of better approximating the dielectric properties found in the literature [22–25]. The phantoms without carbon additives were transparent, which allowed us to later add dyes for the visualization of micro damages and perforations caused by electric current pulses. For dyeing, all transparent head phantoms were placed completely in an emulsion of 1% ironhexacyanoferrate and deionized water for two days after the tests. Table 1 shows the dielectric properties for both types of head phantoms.

**Fig 2:**
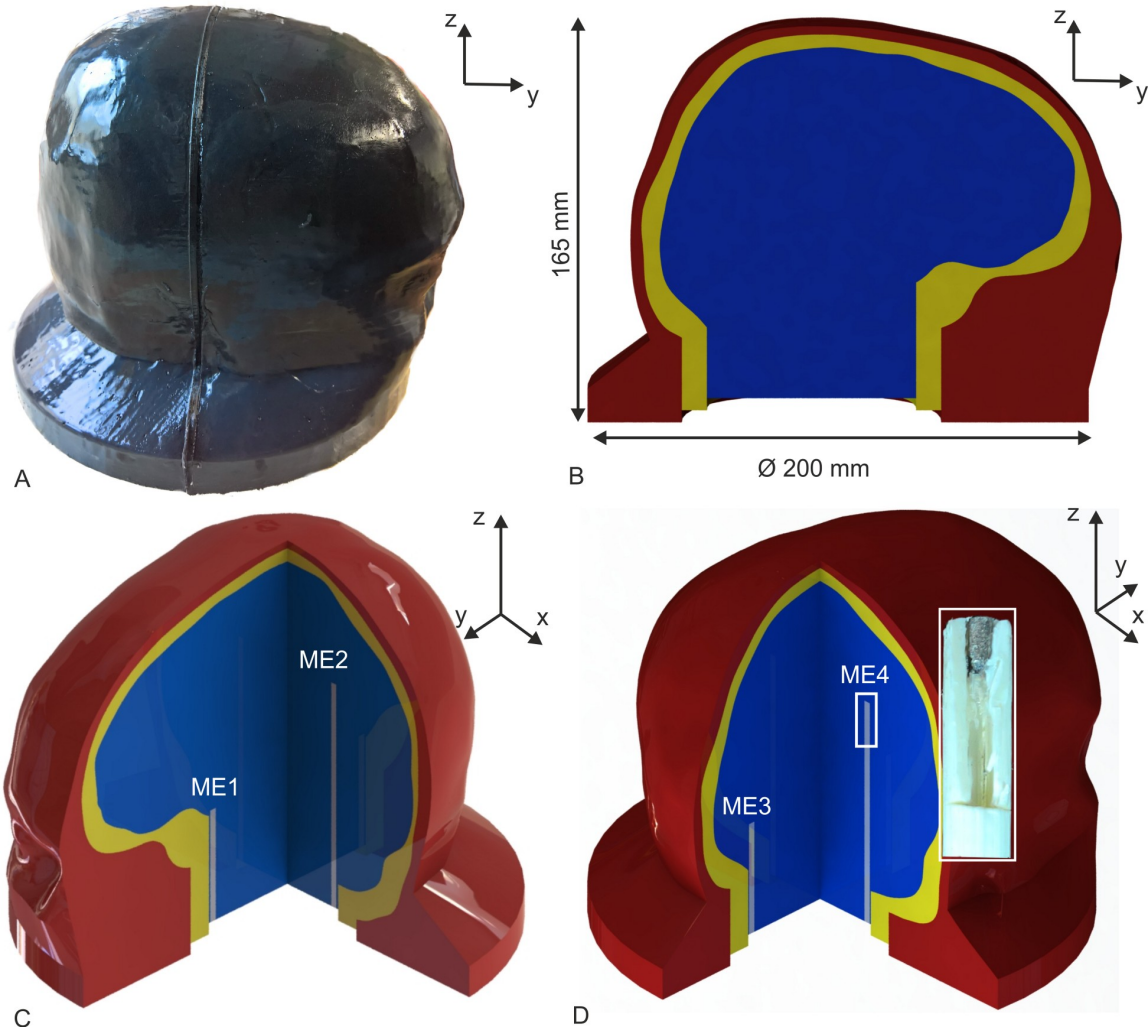
Head phantom geometry and electrode setup of microelectrodes. (A) Formed scalp in a lateral view of a head phantom with carbon additives (head phantom Type I). (B) Sagittal cut through the head phantom. The intracranial volume (brain) is coloured blue, the neurocranium (skull) yellow, and the scalp red. (C) Double cut of the head phantom Type III with the microelectrodes ME1 and ME2 and their holders. (D) Double cut of the head phantom Type III with the microelectrodes ME3 and ME4 and their holders. The inset in D shows a photograph of a microelectrode (grey) with a physical cut through a holder (white) and the wire insulation (yellow).

Fig 2A shows the scalp from head phantom Type I and Fig 2B depicts the structure of head phantoms in a CAD model. The scalp compartment appears black due to the carbon additives. The maximum diameter of each phantom is 200 mm and the maximum height is 165 mm. Furthermore, we created a third type of head phantom (head phantom Type III) which was built up like head phantom Type I but with the addition of four silver/silver-chloride microelectrodes (diameter: 1 mm, length: 2.5 mm, BMP1 from MedCat GmbH, Munich, Germany) in the brain compartment. Thus, it was possible to measure voltages inside head phantom Type III during an applied pulse. The microelectrodes were plugged in holders, which were produced in a fused deposition modelling process and were made of Lignin. Each holder had a diameter of 3 mm. Two holders had a length of 50 mm and the other two had a length of 100 mm. We named the anterior microelectrode ME1, the left microelectrode ME2, the posterior microelectrode ME3, and the right positioned microelectrode ME4 (Fig 2C and Fig 2D).

**Fig 3:**
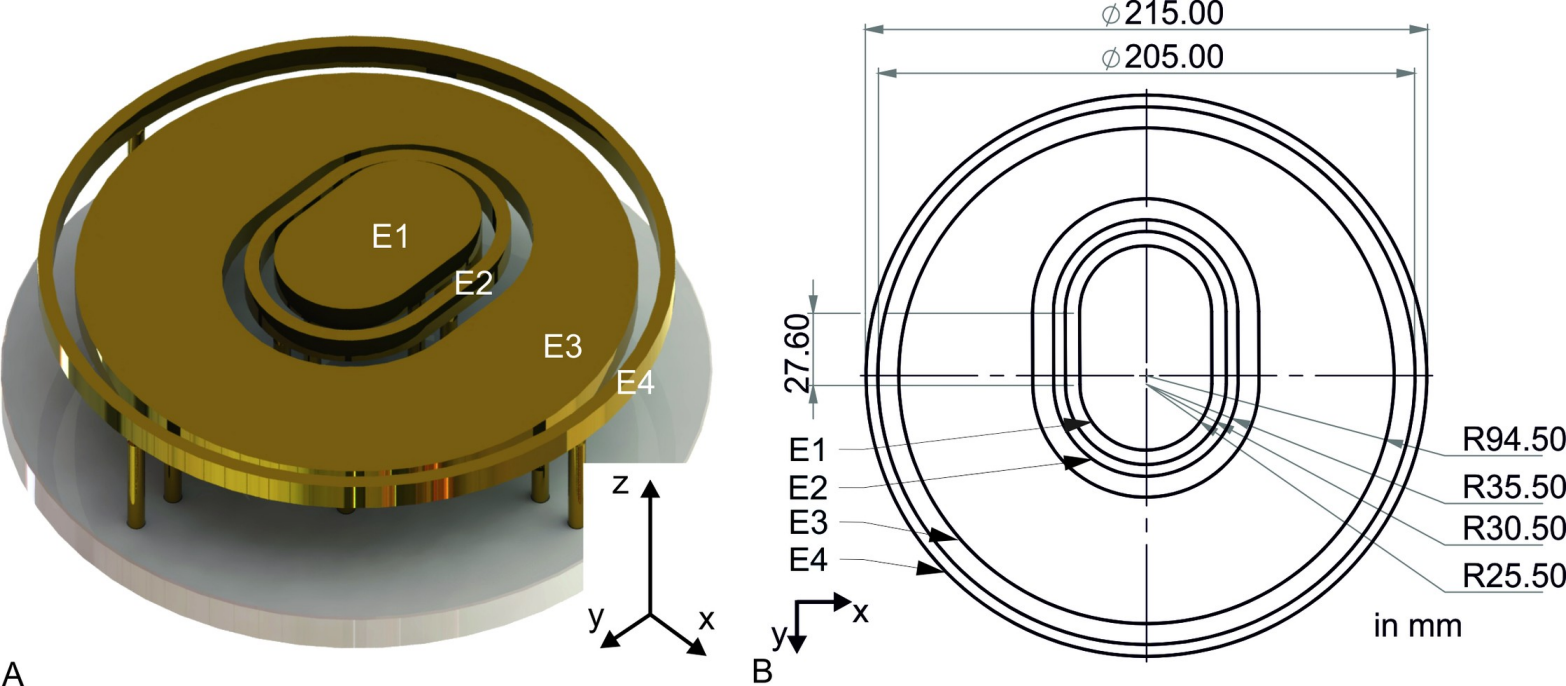
Electrode setup. (A) Schematic of electrode setup with electrodes innermost E1 to outermost E4. (B) Dimensions of the electrode setup in a top view. Each electrode E1–E4 has a thickness of 10 mm.

We created an electrode setup with four separate brass electrodes to detect the current distribution across the compartments of the head phantom. These electrodes were grounded in all the experiments. The geometry of each electrode was adapted to the base area of the compartments to minimize the current path from adjacent compartments. When the head phantom was placed on the electrode setup, the innermost electrode (E1) contacted the brain, the second electrode (E2) the skull and the third electrode (E3) the scalp. The fourth electrode (E4) concentrically surrounded the base of the phantom in a distance of 5 mm (E4: inner diameter 205 mm, outer diameter 215 mm). The chosen dimensions of electrode E4 and the distance to E3 allowed to determine the current distribution in the flashover separately since the ends of the flashovers hit only electrode E4. Flashovers are discharges that start from an impact point on the surface of the head phantom and propagate to the electrode ring E4. These events were detected by a single lens reflex (SLR) camera (EOS 5D Mark II, Canon Inc., Tokyo, Japan). Fig 3 shows the electrode setup and its dimensions.

### Experiments with Marx generator

We used a Marx generator to replicate the high-voltage effects on the human head during a lightning strike. The Marx generator (MG, HIGHVOLT Prueftechnik Dresden GmbH, Germany, properties of MG: Energy 6 kJ, max. charging voltage 125 kV per stage) produces a standardized 1.2/50 μs voltage pulse and can reach a total load voltage of 450 kV [26,27]. An MG can reach a high voltage at the output with one pulse, making it possible to ionize the air over long distances. The discharge is more like a real lightning strike discharge, but the current level of the generated pulse is much lower.

In the first test series, one head phantom Type I was placed on the electrode setup E1–E4 underneath the output from the MG. The distance between the high voltage point electrode (output) and the highest point of the head phantom (vertex) was 150 mm. We applied 15 single positive voltage pulses to the head phantom, each pulse with a total load voltage from the MG of 450 kV. An SLR camera documented the expansion of the lightning discharge. In a second test series, we placed a head phantom Type II on the electrode setup (E1–E4) underneath the output of the MG in the same distance of 150 mm. Again, 15 single voltage pulses were applied, each pulse with a total load voltage of 450 kV. The head phantom Type II was dyed before slicing, as explained in Section: Creation of head phantom. We examined and sliced both head phantoms after the test series. For slicing, we used a strained wire with a diameter of 0.5 mm. The slicing always started with a sagittal cut through one of the observed perforations on the surface of the head phantoms.

### Measurements with combination wave generator

A head phantom Type III was tested with an in-house combination wave generator (CWG) to investigate the electrical behaviour of the head phantom and measure the potential distribution in the brain compartment. The used CWG had the following parameters: Energy 350 J, V_max_ 12 kV 1.2/50 μs, I_max_ 6 kA 8/20 μs [26]. The head phantom was placed on the electrode setup (E4 was decoupled since no flashovers were expected) and a brass electrode (diameter: 25 mm, height: 24.9 mm) was galvanically coupled to the top of the head phantom, near the vertex. This brass electrode was necessary for applying the pulse to the head phantom, because the voltage of the CWG is not high enough to break through larger air gaps like the Marx generator. Further, the influence of an air gap was avoided in the tests to get the electrical behaviour of only the head phantom.

**Fig 4:**
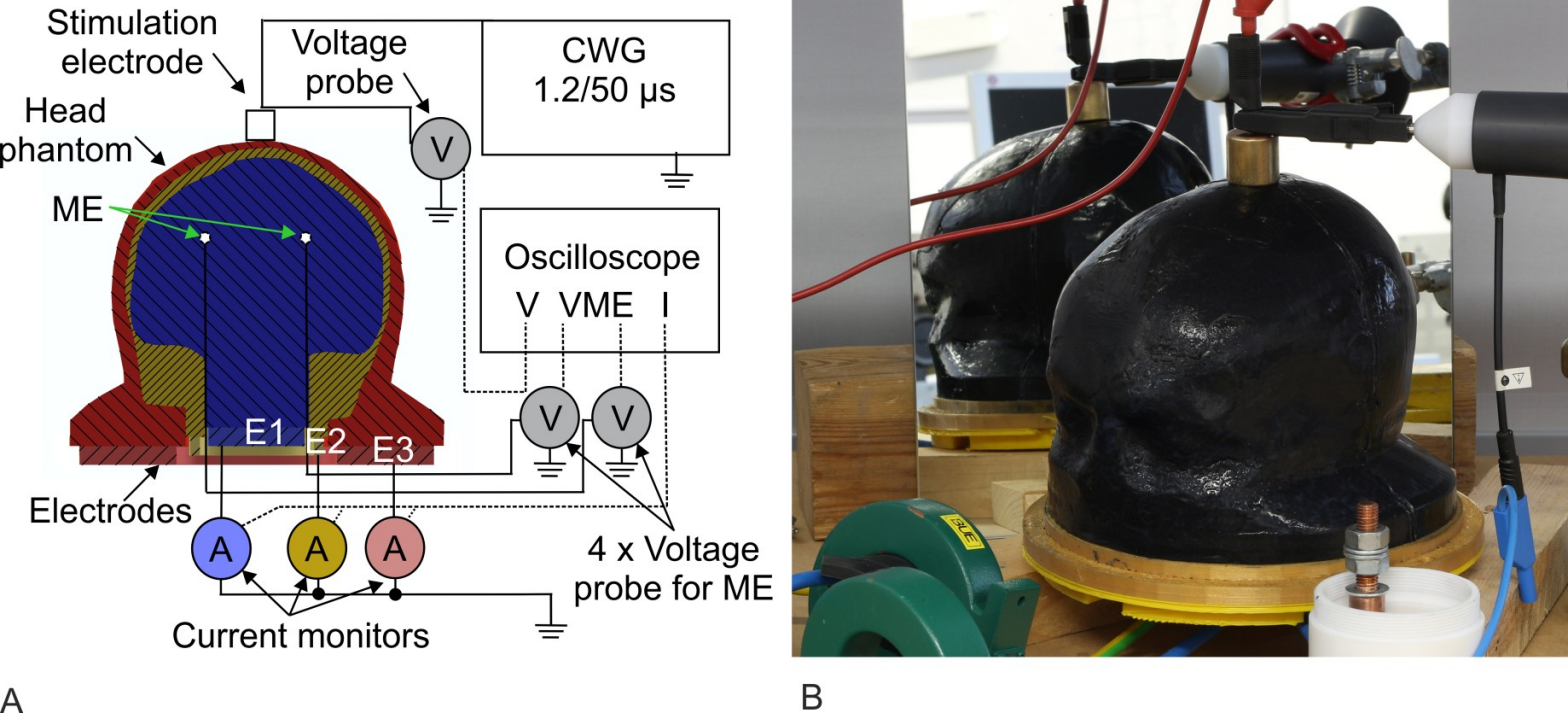
Measurement setup for the experiments with the combination wave generator (CWG). (A) Interconnected measuring equipment with the combination wave generator, head phantom, and oscilloscope. E1 to E3 are the grounded electrodes, each with a current monitor. The four microelectrodes (ME1 to ME4) in the brain compartment were connected by four voltage probes (only two of four probes are symbolized in the schematic). The dashed lines symbolize the measurement wires to the oscilloscope. (B) Photograph of the head phantom Type III placed at the electrode setup and connected via the stimulation electrode to the CWG. A mirror shows the backside of the head phantom Type III.

For the measurements, two Pearson current monitors Model 101 and one Pearson current monitor Model 110 (Pearson Electronics Inc., Palo Alto, CA, United States) detected the currents of E1 to E3, while one voltage probe PHV4002-3 (PMK GmbH, Kassel, Germany) detected the voltage drop over the head phantom. Four voltage probes PHV 1000 (PMK GmbH, Kassel, Germany) recorded the potentials of each microelectrode in the brain compartment. All signals were measured simultaneously by an eight-channel oscilloscope DLM4000 (Yokogawa Electric Corp., Musashino, Japan). We applied 10 positive voltage pulses with an amplitude of 5 kV to the head phantom. The measurement setup is shown in Fig 4. After that, we applied 10 positive voltage pulses with an amplitude of 12 kV to the head phantom. In this test series, the four voltage probes PHV 1000 were disconnected due to their limited admissible voltage of 6 kV. Possible discharges were documented with the SLR camera.

### Measurements with 10/350 μs current pulse generator

We tested four head phantoms on a 10/350 μs current pulse generator to emulate the high-current effects of a direct lightning strike to the head [18]. A current pulse generator reaches a higher current level, different from that obtained by combination wave generator or Marx generator. Therefore, a more realistic lightning current level could be applied to the tested head phantoms. Distances between the output of the generator and the head phantoms were restricted due to the lower voltage. Therefore, the experiments replicated the impact point of a lightning discharge on the head without possible upward connecting lightning leaders.

**Fig 5:**
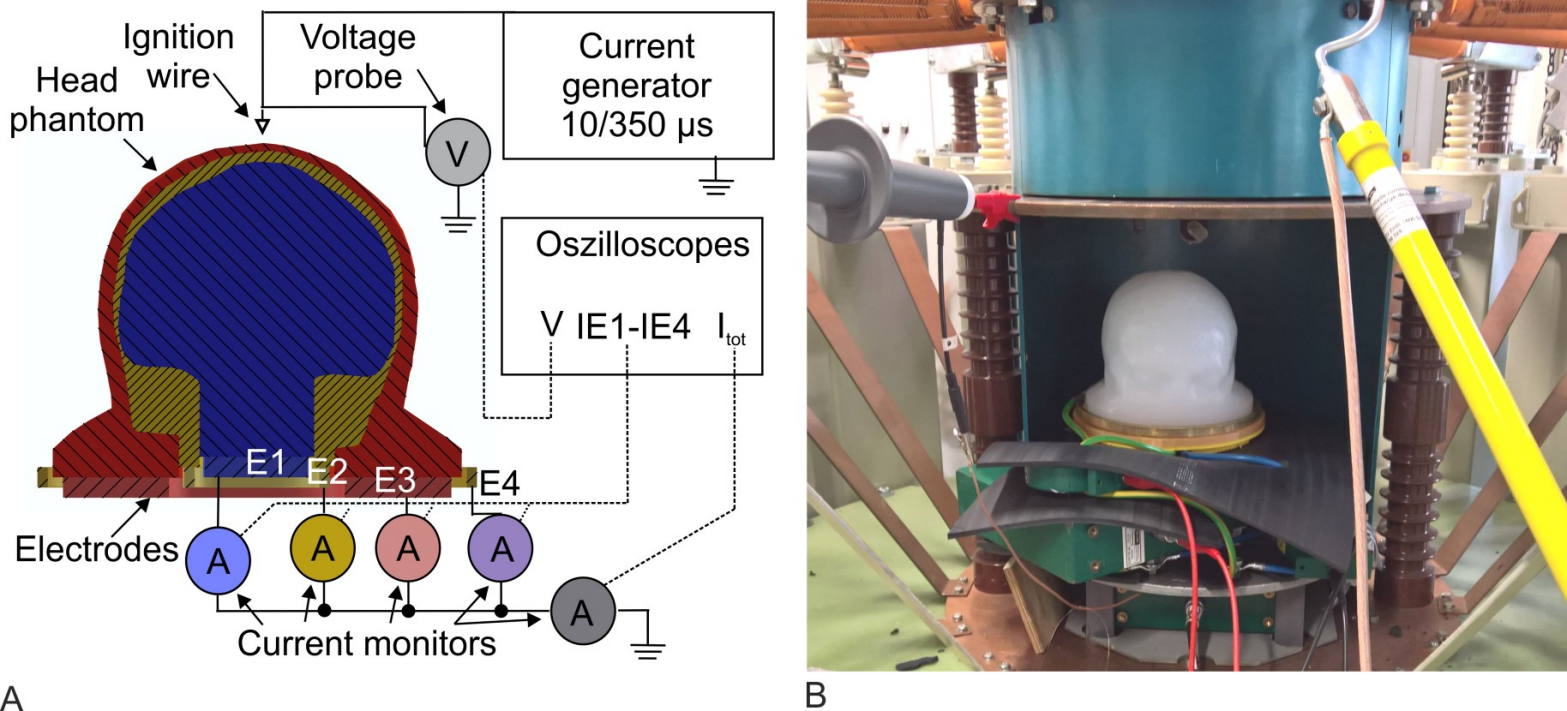
Measurement setup on the 10/350 μs current pulse generator. (A) Schematics of the setup. E1 to E4 are the grounded electrodes. They are connected to the current monitors. The dashed lines indicate the measurement wires to the oscilloscope. (B) Photograph of the head phantom Type II placed in the examination chamber of the current pulse generator.

The generator (IP176/12S of HIGHVOLT Prueftechnik Dresden GmbH, Dresden, Germany) uses 12 switchable single capacitors (called HV-CAP) IS-Phr 12 (VISHAY Electronic GmbH, Selb, Germany). Each HV-CAP has a capacitance of 175 μF and can be loaded with a voltage of up to 12 kV, which results in an energy of 12.6 kJ per charged capacitor. Each head phantom was placed with the electrode setup in the examination chamber of the current pulse generator. The distance from the power output electrode of the current pulse generator to the surface of the head phantom was 55 mm. Therefore, it was necessary to bridge the distance with a copper ignition wire with a diameter of 0.1 mm, which ended about 4 mm above the surface of the head phantom at the vertex. We used three current monitors with a transmission ratio of 1:1000 (Model 1423 Pearson Electronics Inc., Palo Alto, CA, United States) and two current monitors with a transmission ratio of 1:100 (Model 101) to capture the currents in each compartment of the head phantom. The voltage probe (PHV4002-3) detected the voltage drop from the output of the current pulse generator to the ground. All signals were simultaneous recorded from parallel triggered oscilloscopes 44MXI-A and HDO6054 (Teledyne LeCroy, CA, USA). Fig 5 shows the measurement setup for the tests with the 10/350 μs current pulse generator.

We performed different measurement series with this setup on the current pulse generator by changing the number of HV-CAPs to vary the resulting current. Four head phantoms were examined and sliced after the measurements in order to investigate the optically visible damages. In addition, the head phantom Type II was dyed with ironhexacyanoferrate to highlight perforations and damages.

- Test series I: The series started with 12 HV-CAPs @ 12 kV. The number of the HV-CAPs was reduced by three after three applied pulses to the phantom Type 1. At three used HV-CAPs, the number was reduced to one HV-CAP. The charging voltage was always constant. In the last step, the charging voltage was reduced (one HV-CAP @ 6 kV). The used HV-CAP numbers were: 12 @ 12 kV, 9 @ 12 kV, 6 @ 12 kV, 3 @ 12 kV, 1 @ 12 kV, and 1 @ 6 kV. This test series was performed to determine the electrical behaviour and current distribution of the head phantom at different current levels while the positive 10/350 μs pulse.
- Test series II: Repeat of test series I with a newly casted head phantom Type I.
- Test series III: A single pulse with 12 HV-CAPs and a charging voltage of 12 kV was applied to one head phantom Type II. This test was performed to find potential damages and perforations at the transparent head phantom Type II which are not visible with the head phantom Type I.
- Test series IV: The series was performed with a head phantom Type I similar to I and II, but the sequence for the number of used HV-CAPs was pseudo-randomized, starting with six HV-CAPs @ 12 kV.

## Results

### Results of experiments on Marx generator

**Fig 6:**
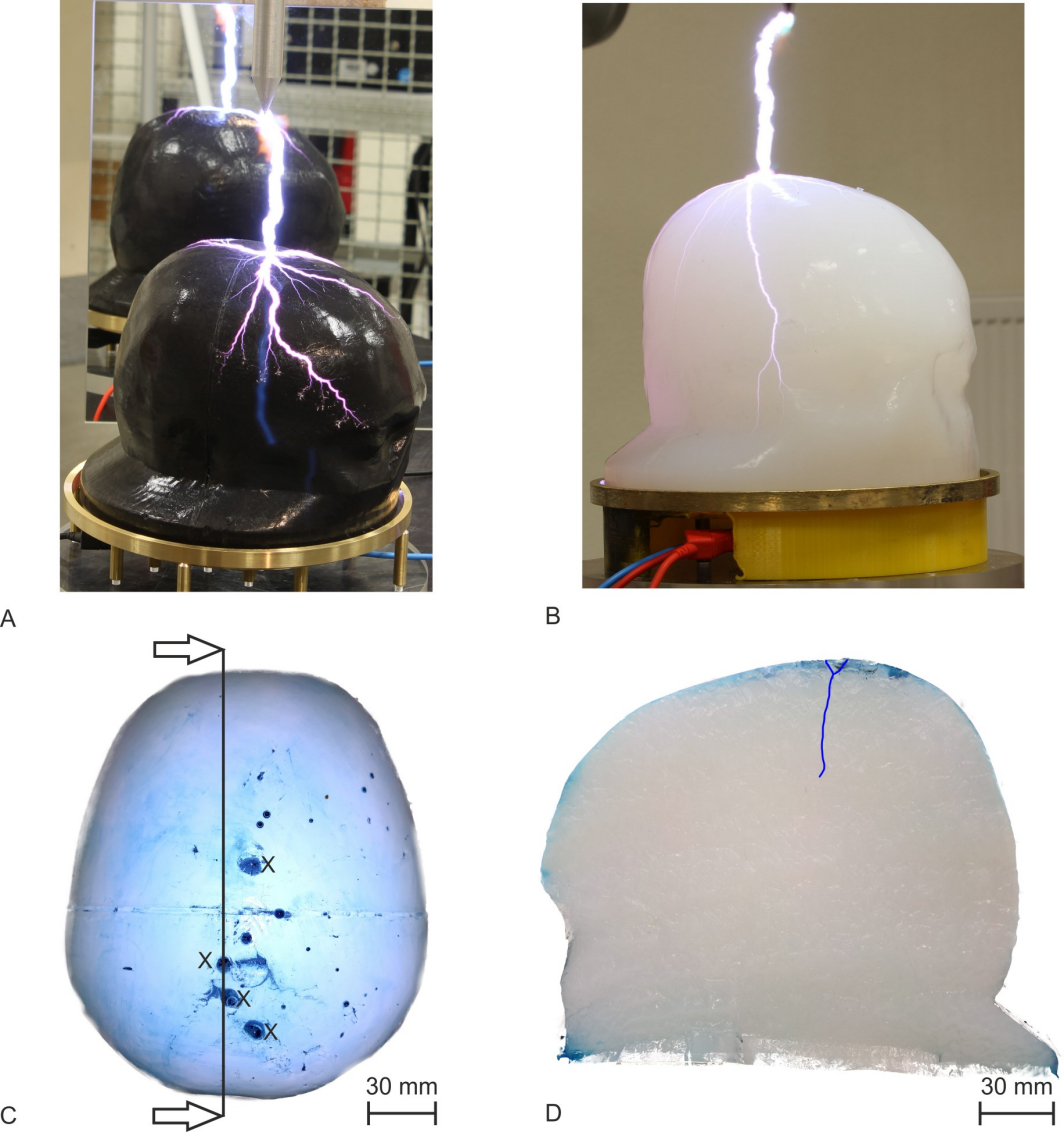
Results of the experiments with the Marx generator. (A) Head phantom Type I and a discharge over the surface of the scalp. A mirror behind the head phantom Type I shows possible discharges on the side facing away from the SLR camera. (B) A discharge over the surface of the scalp from the head phantom Type II. (C) Top view on the scalp compartment from the head phantom Type II after 15 applied single voltage pulses. We marked the biggest perforations with X. The cut line for the first slice is marked black. (D) Sagittal cut along the black line in C, the arrows in C show the view direction. We found a tubular perforation, highlighted in blue colour.

We observed discharges on the surface of the head phantom Type I and head phantom Type II for each 450kV voltage pulse applied from the Marx generator, as shown in Fig 6A and 6B. It can be seen that the discharge channel hit the head phantoms and branched in discharges. The spilt began either at the surface of the head or in the air gap. After the tests, we examined the head phantoms. The head phantom Type I showed a dried ellipsoidal area around the vertex with a length of 120 mm in the anterior–posterior direction and 100 mm in the lateral direction. Also, we found punctured regions with diameters of 2–5 mm. No further particularities were observed in the brain or skull compartment after the slicing of the head phantom Type I. Several perforations occurred in the vicinity of the optically detected impact points at the head phantom Type II. The four largest ones had a diameter of 3–6 mm (marked with X Fig 6C). We sliced the head phantom Type II in a sagittal cut through one impact point, as marked in Fig 6C, and found a dyed channel, which started at the impact point and ended after a length of approx. 50 mm in the brain compartment (highlighted with blue colour in Fig 6D). This perforation was the only one penetrating the brain compartment—all other observed penetrations ended in the skull compartment. There was no systematic difference in the results from head phantom Type I and head phantom Type II. In a previous study, we measured currents of about 2 kA in a simplified head phantom [28] originating from the same pulses of the Marx generator. For the present study, we estimated similar currents in the head phantom Type I and Type II.

### Results of measurements on combination wave generator

**Fig 7:**
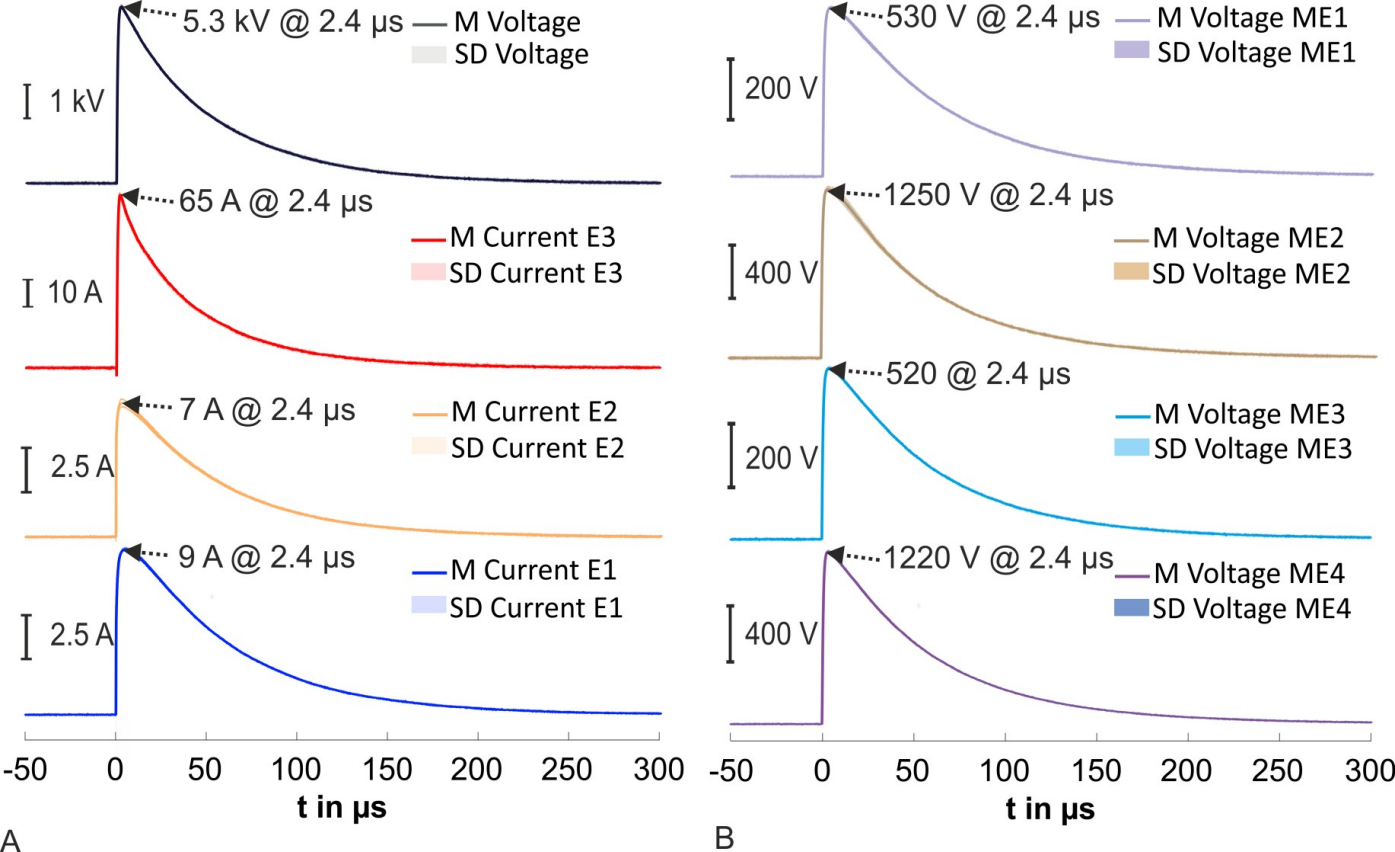
Results of measurements on the combination wave generator with an applied voltage of 5 kV. (A) Average time courses of recorded voltage and measured currents at the electrodes E1 to E3. (B) Time courses of the recorded voltages for the four microelectrodes (ME) in the brain compartment.

No flashover or discharges on the surface of the head phantom Type III were optically detected in the experiments with the CWG and an applied 1.2/50 μs voltage pulse of 5 kV. Fig 7A shows the average recorded time courses of the voltage and the currents of E1 to E3. The mean values at 2.4 μs are additionally indicated in Fig 7A. We calculated the relative current distribution of each compartment with the values at 2.4 μs (maximum measured voltage). The brain was exposed to 11.2%, the skull to 8.6% and the scalp to 80.2% of the current. An average voltage of 530 V was detected at the first microelectrode ME1 at 2.4 μs. Further, the voltage reached 1250 V at ME2, 520 V at ME3, and 1220 V at ME4 (Fig 7B). We calculated the electric field strength (based on the recorded electric potential at 2.4 μs and the distance of the microelectrode to the ground) at these four points: ME1: 10.6 kV/m at ME1; ME2: 12.5 kV/m; ME3: 10.4 kV/m; ME4: 12.2 kV/m.

**Fig 8:**
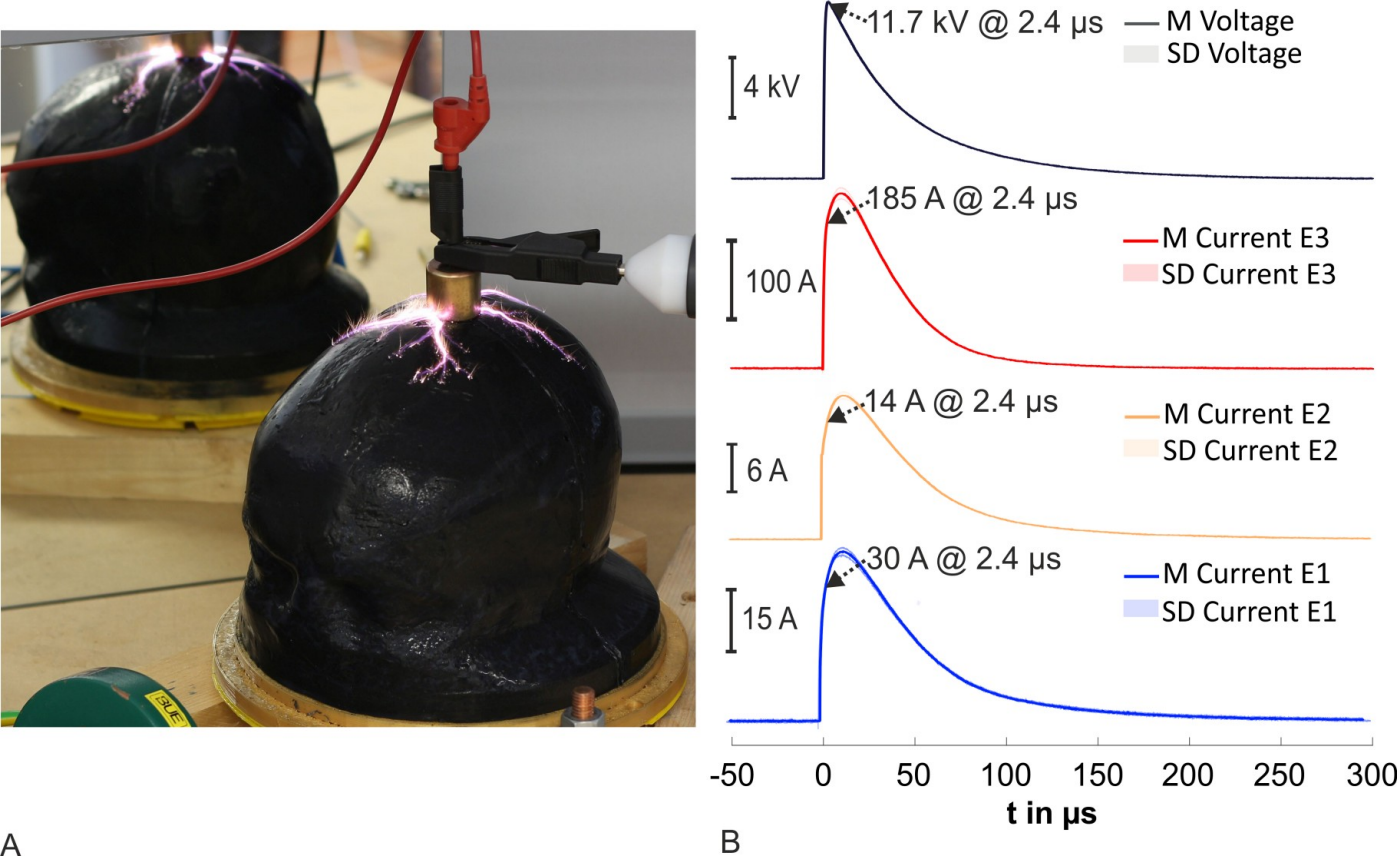
Results of measurements on the combination wave generator with an applied voltage of 12 kV. (A) Registered discharges at the surface of the scalp compartment. The mirror in the background shows the backside of the head phantom. (B) Average time courses of recorded voltage at the generator output and currents from E1 to E3.

We optically detected multiple discharges at the surface of the scalp compartment with an applied voltage of 12 kV (Fig 8A). The discharges started from the attached stimulation electrode. Fig 8B depicts the average recorded time courses of the voltage at the generator output and currents from the electrodes E1 to E3 with labelled mean values at 2.4 μs (maximum measured voltage). The relative current distribution at 2.4 μs was: brain compartment 13.1%, skull compartment 6.1% and scalp compartment 80.8%.

### Results of measurements on 10/350 μs current pulse generator

**Fig 9:**
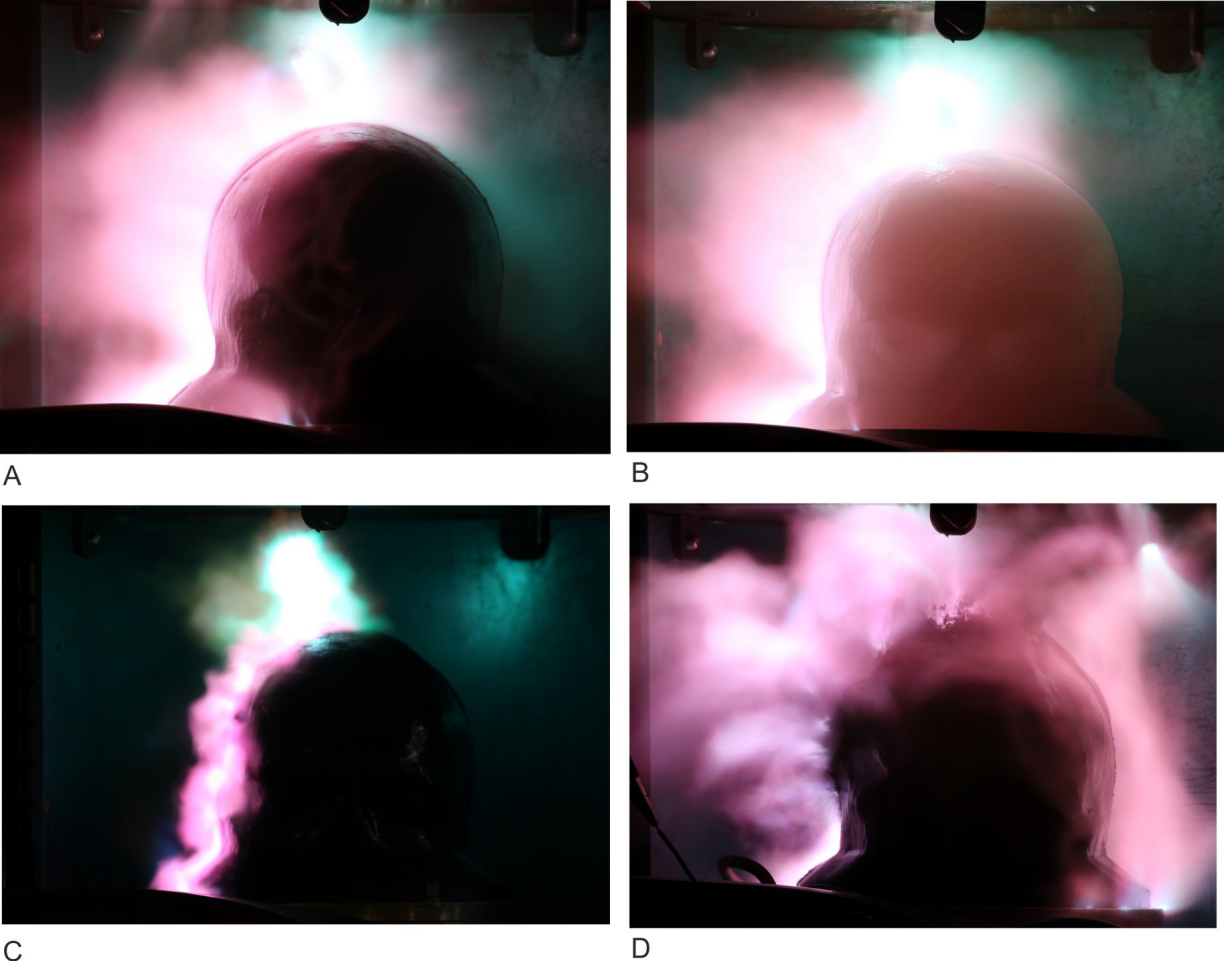
Detected flashovers across head phantoms for the tests with the 10/350 μs current pulse generator. (A) An example of the test series I with 12 HV-CAPs @ 12 kV and with a head phantom Type I. (B) An example of test series III with 12 HV-CAPs @ 12 kV and with head phantom Type II. (C) An exemplary discharge of the test series I with one HV-CAP @ 6 kV and with head phantom Type I. (D) An exemplary discharge of the test series IV at 12 HV-CAPs @ 12 kV with head phantom Type I. The head phantom Type I was destroyed at this discharge.

In each test series, we optically detected a flashover across the head phantoms in the measurements with the 10/350 μs current pulse generator. In almost all tests, the discharge sat on near the highest point of the head phantoms (vertex) and went over the scalp to E4. Fig 9 depicts four different morphology examples. Fig 9A and 9B show exemplary discharges of the test series I and III with 12 HV-CAPs @ 12 kV with a head phantom Type I and the head phantom Type II, respectively. In both cases, the discharge channel appeared structured in the vicinity of the scalp. However, the discharge channel was surrounded by a corona. The discharge and the resulting flashover were less intensive and the corona around the channel was smaller with one HV-CAP @ 6 kV in contrast to 12 HV-CAPs @ 12 kV. An example of a discharge with one HV-CAP @ 6 kV is shown in Fig 9C. Fig 9D depicts the last applied current pulse of the test series IV (after three times six HV-CAPS @ 12 kV and one time 12 HV-CAPS @ 12 kV). The main discharge over the scalp and the discharge channel are difficult to identify. Discharges next to the scalp appeared especially on the right side of the head phantom Type I. The discharges busted the phantom material at several points.

All recorded voltages and currents showed characteristic time courses. The voltage increased up to the load voltage in about 1 μs; simultaneously, the current increased through the head phantom. Then the voltage decreased rapidly to a value of about 1.5 kV in approximately 50 μs. At the same time, the current in E4 increased and showed an aperiodic pulse behaviour.

**Fig 10:**
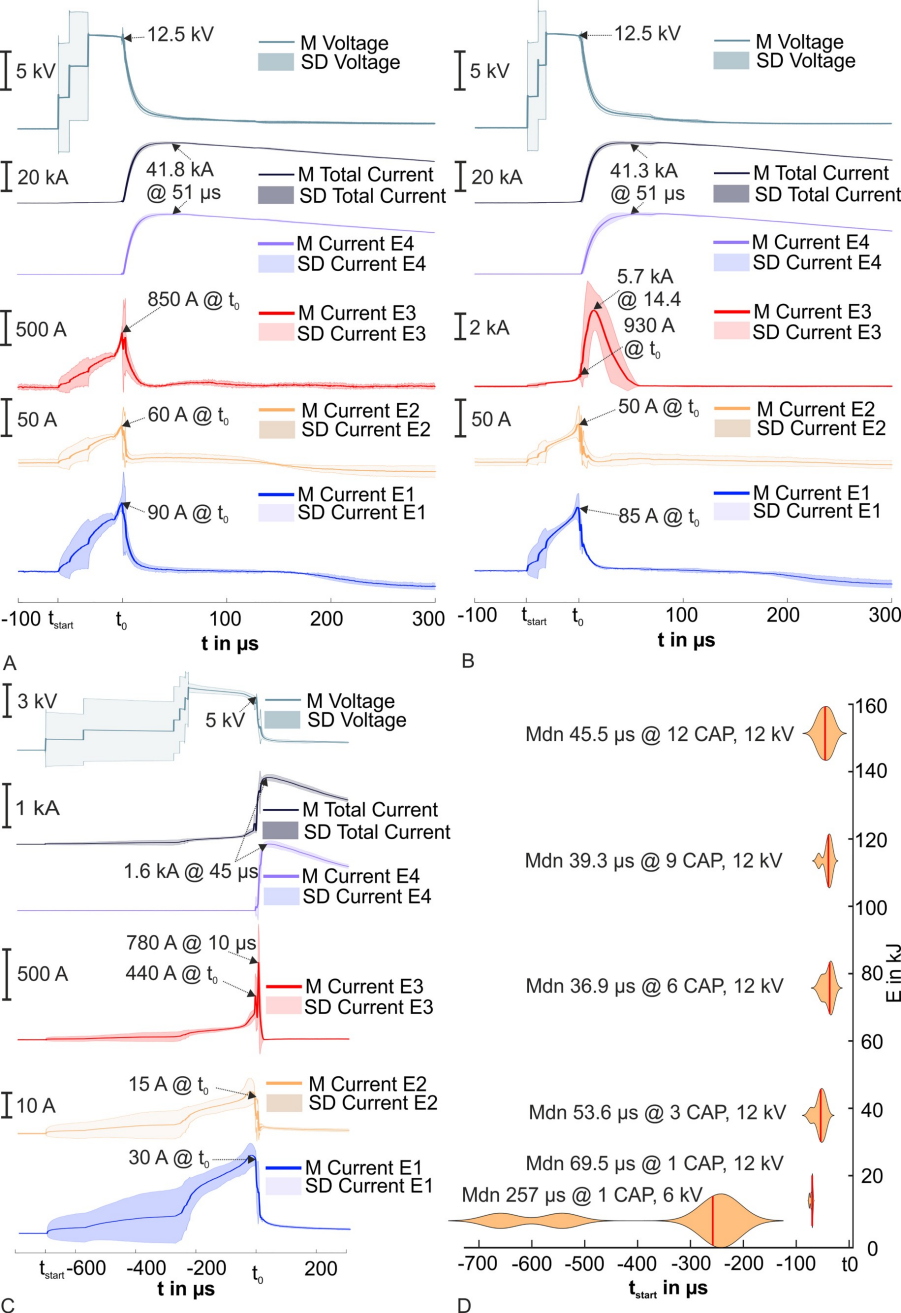
Time courses from test series I and II with the 10/350 μs current pulse generator. (A) Time courses for 12 HV-CAPs @ 12 kV for an example Case 1 at test series I and II. (B) Time courses for 12 HV-CAPs @ 12 kV for an example Case 2 at test series I and II. (C) Time courses for one HV-CAP @ 6 kV. (D) Violin plots of t_start_ for each tested capacitor stage (applied energy). The time t_start_ was defined as latency at which we registered a first appearance of voltages or currents different from zero. The time to start of the flashover was called t_0_. M: mean, Mdn: median, SD: standard deviation.

Fig 10A and 10B present the average time courses of the test series I and II at 12 HV-CAPs @ 12 kV with the most important key values. It can be seen that simultaneous to the first rise of the voltage, the current in E1, E2, E3 and the total current also increase. The time point t_start_ indicates the beginning of the increasing voltage and current. The discharge process started over the head phantom at this time point. After the increase of the current in E1 and E2 to the maximum, these currents broke down rapidly, along with the voltage and the current in E3 (Case 1). We defined this point as the moment of the beginning of the evolvement of the complete flashover across the head phantom and labelled it t_0_, determined by the highest decrease speed with the first derivation of the voltage. After the rapid collapse of the current in E1 and E2 close to zero ampere, the current in E4 increased from zero ampere to a high amplitude (in Fig 10A and 10B, up to 41.8 kA or 41.3 kA). Furthermore, we found a second possible case (Case 2) for the current time course in E3. The current in E3 broke in first, increased again, and reached a multiple higher peak, for example from 930 A at t_0_ to 5.7 kA after further 14.4 μs. This case is shown as an example in Fig 10B. Fig 10C depicts the time courses from the test series I and II at one HV-CAP @ 6 kV. The duration to t_0_ increases in comparison to the time courses in Fig 10A and 10B, but the time course shows some similarity in shape. The applied pulses ended about 2.6 ms after t_0_. As a further result, we extracted the time points t_start_ for each test for the test series I and II. The distribution is shown in Fig 10D. The time of starting of the flashover (t_start_) for the head phantom was in median between 40 μs and 45 μs at an energy level between 151.2 kJ and 75.6 kJ. The time increased from the level of 37.8 kJ at 53.6 μs over 12.6 kJ at 69.5μs to 6.3 kJ at 257 μs. The time t_start_ differed most at 6.3 kJ between 219 μs and 694 μs.

Table 2 provides the calculated relative current distribution for the compartments at t_0_ and at the latency of the maximum of the total current after the fully formed flashover (t_maxtot_), based on the values (test series I and II) of the currents from each tested capacitor stage. The skin compartment was most exposed to the applied current at t_0_ for each used HV-CAP stage, followed by the brain compartment and the skull compartment. After a flashover, the highest currents were detected at t_maxtot_ in electrode E4 for each capacitor stage; we found fractions of current between 98.2% and 99.5% in E4. The next highest part flowed through the scalp compartment. Brain and skull compartments had a smaller exposition, each with a current below 7 A. For the brain and skull compartments, the relative current distribution increased with a lower energy level at t_maxtot_.

**Table 2:** Relative current distribution for the test series I and II at the time points before (t_0_) and after a fully formed flashover (t_maxtot_).

HV-CAPS charged up to 12 kV	Total energy in kJ	Applied max. current @ t_0_ in kA	Applied max. current @ t_maxtot_ in kA	Rel. current distribution for flashover in %		Rel. current distribution for scalp in %		Rel. current distribution for skull in %		Rel. current distribution for brain in %	
				t_0_	t_maxtot_	t_0_	t_maxtot_	t_0_	t_maxtot_	t_0_	t_maxtot_
**12**	151.2	1.00	41.5	n/a	99.5	85.8	0.45	5.1	<0.02	9.1	<0.02
**9**	113.4	0.95	31.8	n/a	99.5	87.2	0.32	4.6	<0.02	8.2	<0.02
**6**	75.6	1.40	21.5	n/a	99.5	90.4	0.38	3.5	<0.02	6	<0.02
**3**	37.8	0.77	10.9	n/a	98.2	83.5	1.7	5.9	0.05	10.6	0.06
**1**	12.6	0.97	3.7	n/a	98.9	87.2	0.8	4.3	0.08	8.5	0.2
**1 (6 kV)**	6.3	0.48	1.55	n/a	99.2	90.5	0.3	3.1	0.2	6.4	0.3

**Fig 11:**
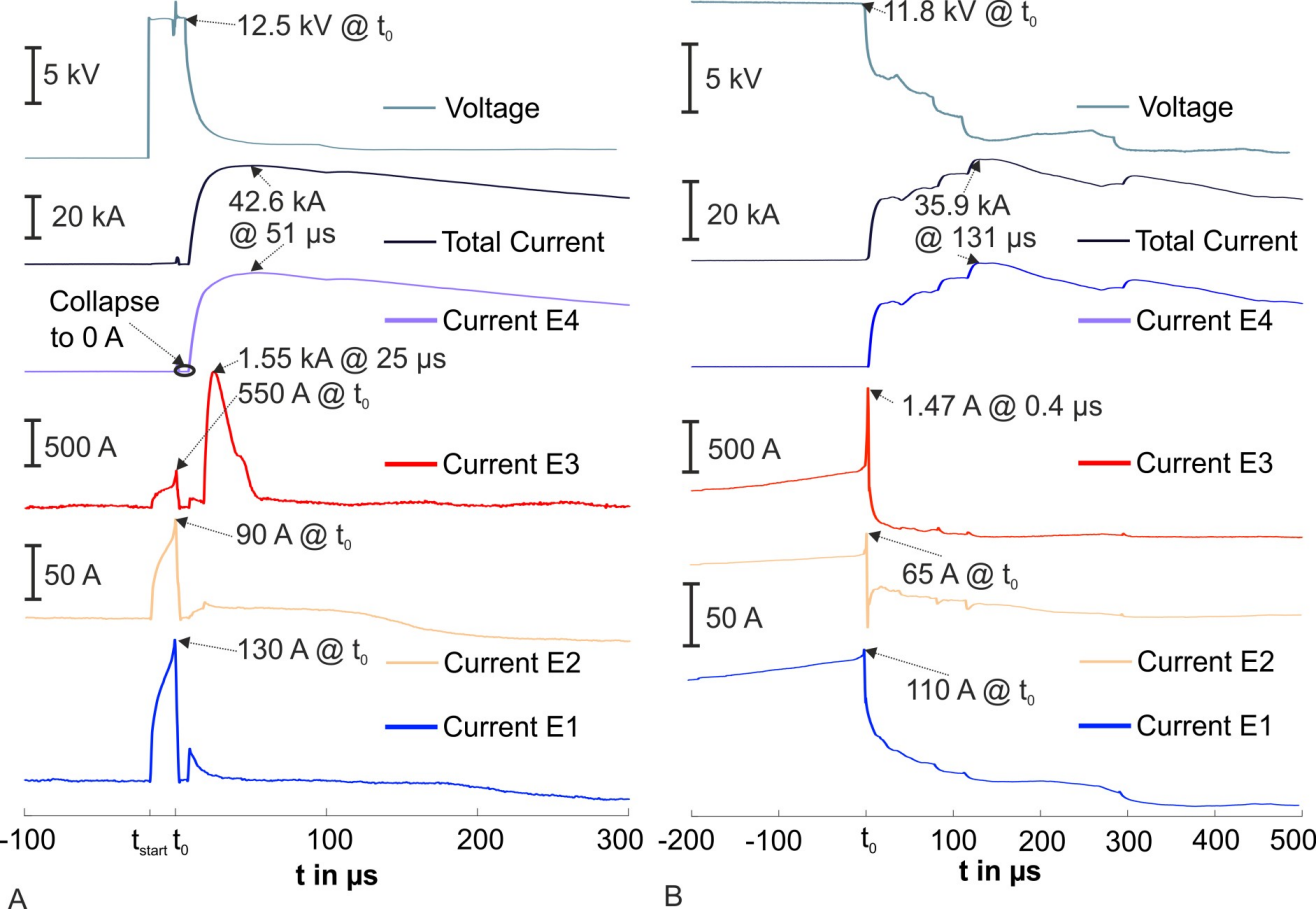
Time courses from test series III and IV with the 10/350 μs current pulse generator. (A) Shows the time courses for the test series III, the only one test series with the head phantom Type II and 12 HV-CAPs @ 12 kV. (B) Depicts the recorded time courses for the test series IV at the second repeat with 12 HV-CAPs @ 12 kV.

The time courses of the currents in test series III were also aperiodic, as in test series I and II for case 2, but we found a second increase in the current in E3, probably caused by an incomplete flashover. The currents in E1, E2, and E3 decreased and the current increased up to approximately 2.5 kA in E4 after the breakdown of the voltage. After that, we registered an increase of the voltage, an increase of the currents at E1–E3, and a collapse to 0 A in E4 (named collapse in Fig 11A). After another 5 μs, the voltage broke down again as well as the currents in E1 to E3. The current in E4 increased and followed the typical aperiodic time course. The measurement of the time courses from test series IV with 12 HV-CAPs @ 12 kV are shown in Fig 11B. The time courses were aperiodic too, but the time to the event points were longer than the measured durations from other test series. We could not record the event t_start_. The moment t_maxtot_ was reached after 131 μs after t_0_. Further, the time course showed waves between t_0_ and t_maxtot_.

The maximum current steepness is a parameter to define and compare lightning current pulses. We calculated the maximum current steepness of the measured total currents with the 30%–90%-rule of amplitude. We used the 30%–90%-rule because the current amplitude at 10% was not measurable [18]. The maximum current steepness showed a reduction from about 1855 A/μs at an applied energy of 151.2 kJ down to 64 A/μs at 6.3 kJ. Compared to the ideal 10/350 μs current pulses (assumed double exponential function), the measured current pulses had a less current steepness with a factor between 0.95 and 2.08, as shown in Table 3. The difference of current steepness between the measured current pulses and the ideal 10/350 μs current pulses is due to impedances of the head phantom and the discharge channel. The 10/350 μs current pulse generator is not an ideal constant current source; additional impedances extend the pulse form.

**Table 3:** Current steepness of measured total currents in test series I and II and comparison with ideal 10/350 μs current pulses.

HV-CAPS charged up to 12 kV	Total energy in kJ	Current steepness in A/μs of measured total current pulses	Current steepness in A/μs of ideal 10/350 μs current pulses	Factor between measured and ideal current pulses
**12**	151.2	1855	3557	1.92
**9**	113.4	1501	2726	1.82
**6**	75.6	1260	1843	1.46
**3**	37.8	655	934	1.43
**1**	12.6	334	317	0.95
**1 (6 kV)**	6.3	64	133	2.08

We examined and sliced the head phantoms after the test series, with the following results.

**Fig 12:**
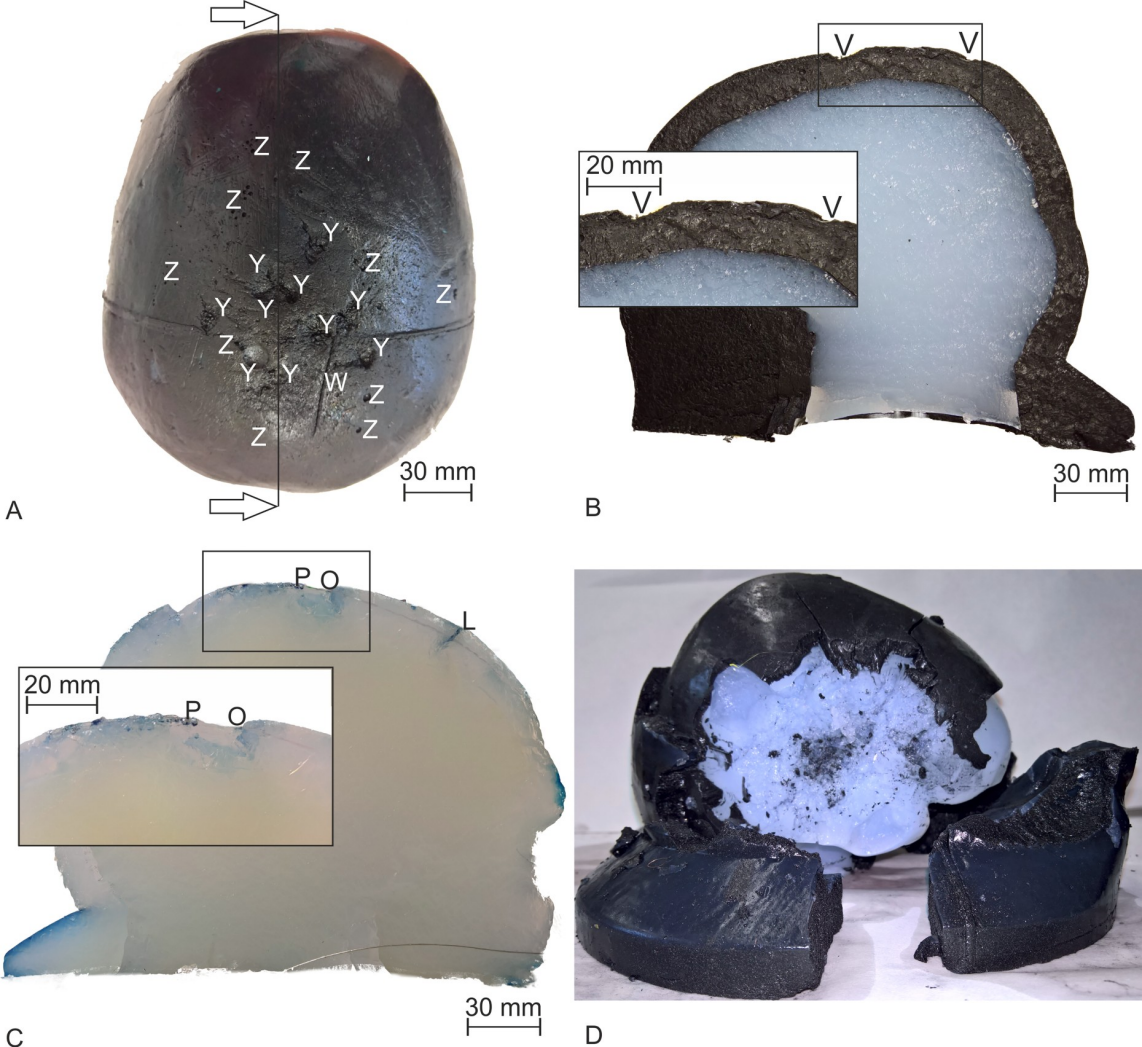
Examined head phantoms after the tests with the 10/350 μs current pulse generator. (A) Damaged and dried areas at the top of the scalp compartment for the head phantom Type I after the test series I. The impact points are marked with Y smaller perforations with Z and a current path with W. The black line marks the cut line. The arrows indicate the view direction for the cut. (B) Slice of the head phantom Type I indicated in A. Burned and perforated areas were magnified in the inset (marked with V). (C) Sagittal sliced head phantom Type II from test series III. A perforation was marked with L. Inset shows magnified area around the impact point O and P mark blistering in the scalp compartment. (D) The destroyed head phantom Type I after the test series IV. The scalp and skull compartments were blown open, and the brain compartment was damaged and melted.

- Test series I and II: We found similar damages on both head phantoms Type I from test series I and II, which are described for one phantom in the following: 10 impact points were found on the top surface of the scalp (Fig 12A), ranging from 5 mm to 13 mm in diameter—they are marked with Y. Several smaller perforations are marked with Z in Fig 12A. One path (outgoing from an impact point) was found in the right parietal occipital region, with a length of about 45 mm (marked with W in Fig 12A). We sliced the head phantoms with a sagittal cut through an impact point. In the sagittal cut of one head phantom Type I (Fig 12B), we noticed damages at the upper part of the skull near the impacts in the form of drying and melting, marked as V. The brain compartment seemed to be unaffected (Fig 12B).
- Test series III: This head phantom Type II was dyed with ironhexacyanoferrate to highlight the damages. We found an ellipsoidal impact point on the scalp near the vertex, with an approx. length of 7 mm from anterior to posterior and 10 mm from right to left. Three obvious burn paths were found to be outgoing from the main impact point. The first path had a length of 110 mm to posterior direction, the second path a length of 95 mm to right parietal, and the third path a length of 80 mm to right parietal occipital. Furthermore, we detected perforations in the upper part of the skull near the impact point. We sliced the head phantom Type II with a sagittal cut through the impact point. One perforation had a length of 10 mm down to the skull, marked with L in Fig 12C. The impact point is marked with O in Fig 12C. We found melting and blistering points around the impact point (marked with P Fig 12C). No optical damages were found in the brain compartment.
- Test series VI: The head phantom Type I (Fig 12D) was destroyed after three pulses applied with six HV-CAPs and two pulses applied with 12 HV-CAPs (in the second applied pulse). The scalp was blown up and the skull was broken on the right side of the head phantom. We found a melted area in the brain compartment under the cracked skull with the dimensions of 120 mm anterior to posterior and 95 mm inferior to superior.

## Discussion

### Spatial current distribution in the head phantom

We obtained the relative current distribution in the compartments of the created head phantoms and in discharge channels during various applied voltage and current pulses. In case of no flashover, the scalp compartment was always most exposed to the current (80–90%) in all experiments. The brain compartment was exposed to 6–13% of the current. The skull compartment experienced the smallest amount of current—about 3–6%. In case of flashover formation, the relative current distribution changed and 98–99% of the current flowed in the discharge channel due to its higher electrical conductivity compared to the head phantom. The remaining 1–2% of the current again predominantly flowed in the scalp, followed by the brain and skull compartments. We observed this distribution pattern (most current in the scalp, followed by brain and skull compartments) across all experiments, independent of the applied voltage and current pulse forms. To the best of our knowledge, this is the first time that a study has reported such current distributions that establish normative values to be used in later experimental investigations on the possibilities of lightning protection equipment for humans.

### Voltage and current time courses

The time courses of the recorded voltages and currents show distinct differences for the cases without and with flashover. For the cases without flashover, we found the expected rise and fall of both voltage and current time courses according to the applied 1.2/50 μs pulse.

For cases with flashover, we found three time intervals: a short rising time of the voltage lasting approximately 1 μs after t_start_ marked the initial interval indicating the connecting leader phase [12]. Thereafter, the time interval until t_0_, the initial return stroke phase [12], is characterized by increasing current in and a constant voltage across the head phantom. We measured a time of approximately 30–700 μs for the second interval. The third interval starts with t_0_ and ends after 2.6 ms and is mostly dominated by the high pulse current; this interval is the denominated surface flashover phase [12,13]. Contrary to the theoretical predictions by Uman [12], our experiments show an almost constant voltage across the head phantom and a nonlinearly increasing current in the head phantom for the first and second time intervals. While Uman [12] used an impressed pulse voltage for the first and second time intervals (t_start_ to t_0_), in our experiments, the 10/350 μs current pulse generator provided an impressed constant voltage during the first and second time intervals.

In one experiment, the head phantom was destroyed by the impressed pulse. In this specific case, in the third time interval, we found time courses for voltages and currents that exhibited a step-like and spiky behaviour (Fig 11B). In this interval, the voltage and current irregularly increased and decreased, possibly indicating a change in the electrical conductivity. We assume that these changes in the electrical conductivity might be caused by thermal ionization and plasma processes directly in the head phantom [29].

### Flashover

The flashover is a phenomenon that occurs in technical setups and during lightning accidents with humans [12,13,29]. In our experiments, the flashover took approximately 30–700 μs to form. Differently, a time of 30–150 ns characterizes flashovers for lightning accidents [13] and a few 100 ns for technical setups [29,30]. The time to form a flashover mainly depends on the voltage amplitude and the pulse form. The observed difference in time were likely caused by the voltage differences: our experiments operated with 12 kV while a lightning strike has about 1 MV [11,13]. Bazelian and Raizer characterize times up to 200 μs for fully formed discharge channels along water surfaces [31]. This implies that the time to form a flashover increases with a lower impedance such as biological tissues.

The time to form a flashover increased in median from 69.5 μs at 1 CAP at 12 kV to 257 μs for 1 CAP at 6 kV (Fig 10D). This behaviour is expected for such impulse voltage-time characteristics [29,30].

### Implications for lightning protection

Our experiments confirmed that most of the current (98–99%) flowed outside of the human head in the discharge channel after a fully formed flashover [12,13]. Consequently, the faster a flashover is formed over the head, the higher are the chances of survival. Further, the current flowing in the brain compartment is typically a factor of 20 units less in amplitude compared to the current flowing in the scalp. This difference is caused by the protective property of the skull due to its low electrical conductivity and relative permittivity compared to the scalp and brain compartments [22].

Electroporation can cause cell damage and death [32–35]. An electric field strength of 6 kV/m and above can cause reversible or irreversible electroporation in biological cells [32–35]. An electric field strength of about 100–600 kV/m close to the ground are typical values during lightning [11]. Our experiments show an electric field strength of about 10.4–12.5 kV/m in the brain compartment during an applied 1.2/50 μs voltage pulse with an amplitude of 5 kV. These values indicate that electroporation can occur even for the lower current and voltage amplitudes in our experiments. Thus, neurological damages during a lightning strike caused by electroporation are likely to occur [6,35].

We applied high voltage (Marx generator) and high current pulses (10/350 μs current pulse generator) to different head phantoms and optically analysed the damages to the head phantoms. In all setups, we found similar damages, e.g. perforations and burns on the scalp compartment or dried areas throughout the scalp compartment down to the skull. These observations are similar to autopsy reports after lightning accidents [3,8,15].

According to the DIN EN 1149, a personal lightning protection equipment, for example in the form of a coat with a hood consisting of modacrylics (flame retardant synthetic copolymer), can reduce the current amplitudes in the head compartments due to the material’s electrostatic discharge behaviour [16]. Consequently, this lightning protection equipment can prevent burns and perforation as well.

### Experimental setup and limitations

Physical head phantoms allow us to gain insight into head internal field and current distributions. They can provide explanations for observed lightning phenomena and can help to develop novel hypotheses and experiments. However, physical head phantoms are simplifications. Our head phantoms replicate the three main head compartments in terms of their geometric and dielectric properties, but neglect the highly complex interior structures of the human head, like the sulcus/gyrus structure, the anisotropic conductivity of the white matter, according Haueisen et al. [36], or hair on the scalp. Future research should include more detailed head phantoms.

Another limitation was the measurement setup for the current distribution. The spatial and temporal resolutions of our measurement equipment (oscilloscopes, current monitors, voltage probes) were limited as well. Due to these limitations, we were unable to measure the initial connecting leader phase, in which a negative current typically flows at the start of the discharge.

At present, voltage and current generators cannot provide a high voltage and a high current simultaneously similar to a lightning discharge. For this reason, we use three different generators to replicate the voltage and current effects on the head phantoms. We compare the generators in the following paragraph.

- The Marx generator (MG) generates a 1.2/50 μs high voltage of up to 450 kV. The high voltage ionizes the air gap over a larger distance between the high voltage point electrode and the head phantom, mimicking the air distance between the cloud and the ground during a lightning discharge [27]. The energy and the resulting current (about 2 kA) values are low in comparison to natural lightning. Further, no flashovers are possible due to the low energy and the impedance of the head phantom. The used MG is only suitable for visually investigating the high voltage effects which manifested as perforations on the head phantoms.
- The combination wave generator (CWG) produces a 1.2/50 μs voltage up to 12 kV (open-circuit) or an 8/20 μs current up to 6 kA (short-circuit), depending on the impedance of the test object according to IEC 61000–4–5 [26]. The voltage amplitude was not high enough to ionize the air over larger distances. For this reason, a stimulation electrode galvanically contacted the scalp compartment in our experiments. The low noise and low interference setup enabled a voltage and current measurement with a high signal noise ratio and a high reproducibility in our experiments. This setup also allowed the measurement of voltages at electrodes integrated in the brain compartment (ME1–ME4). Another advantage of the CWG is the similarity of its voltage pulse form to a lightning voltage pulse form.
- The 10/350 μs current pulse generator can be charged up to 12 kV according to IEC 62305 [18]. The maximum current in the experiments was about 42 kA. Due to the low voltage, an air gap of only 4 mm between the output of the generator and the test object was possible. We used an ignition wire to increase this distance to 55 mm. Consequently, no connecting upward leader was formed. The used current amplitude and current steepness corresponded to real lightning currents. For the estimation of the current distribution in the head phantom, including the flashover, the 10/350 μs current pulse generator was best suited for comparison to real lightning currents.

## Conclusion and outlook

We created head phantoms made of agarose to replicate the scalp, the neurocranium, and the intracranial volume of a human head. These head phantoms were tested on pulse generators, which modelled lightning components to investigate the physical and biological effects during a lightning strike. We found:

1. The scalp compartment is most exposed to the current during a lightning strike, with a fraction of approximately three quarters.
2. A fully formed flashover rapidly reduces the current in the brain compartment and the current exposition is reduced in interior structures the faster a flashover is formed. This mechanism can explain why people can survive a lightning strike.
3. The skull has protective properties due to its low electrical conductivity and relative permittivity.
4. The electric field strength in our experiments reaches a level where electroporation is possible. The mechanism of electroporation may explain neurological effects and damages after a lightning strike.

We established normative values in our experiments for the human head to lightning discharges without any protective equipment. In the next steps, we will compare the found values with protective equipment applied to the head phantom. Further, we will improve our head phantoms to be more realistic models (e.g. add hair on scalp compartment).

## References

[1] Cooper MA (1980) Lightning injuries: Prognostic signs for death. Annals of Emergency Medicine 9 (3): 134–138. 10.1016/s0196-0644(80)80268-x 7362103

[2] Elsom DM (1996) Surviving being struck by lightning: A preliminary assessment of the risk of lightning injuries and death in the British Isles. Journal of Meteorology 21: 197–206.

[3] Cooper MA, Andrews C, Holle R, Blumenthal R, Navarrete Aldana N. Lightning injuries. In: Wilderness Medicine; 2016 pp. 60–101.

[4] Cooper MA, Holle RL. Reducing lightning injuries worldwide: Springer natural hazards; 2019.

[5] Andrews CJ, Cooper MA, Darveniza M, Mackerras D. Lightning injuries. Electrical, medical, and legal aspects; 1992.

[6] Andrews CJ, Reisner AD (2017) Neurological and neuropsychological consequences of electrical and lightning shock: review and theories of causation. Neural regeneration research 12 (5): 677–686. 10.4103/1673-5374.206636 28616016 PMC5461597

[7] Freeman CB, Goyal M, Bourque PR (2004) MR imaging findings in delayed reversible myelopathy from lightning strike. AJNR. American journal of neuroradiology 25 (5): 851–853. 15140734 PMC7974502

[8] Mann H, Kozic Z, Boulos MI (1983) CT of lightning injury. AJNR. American journal of neuroradiology 4 (4): 976–977. 6410883 PMC8333752

[9] Stanley LD, Suss RA (1985) Intracerebral hematoma secondary to lightning stroke: Case report and review of the literature. Neurosurgery 16 (5): 686–688. 10.1227/00006123-198505000-00020 4000443

[10] Zack F, Rothschild MA, Wegener R (2007) Lightning strike–Mechanisms of energy eransfer, cause of death, types of injury. Deutsches Aerzteblatt International 104 (51–52): 3545 10.3238/arztebl.2008.0224a

[11] Rakov VA, Uman MA. Lightning Physics and effects. Cambridge, U.K., New York: Cambridge University Press; 2006.

[12] Uman MA. The art and science of lightning protection Cambridge: Cambridge University Press: 2010.

[13] Cooray V. An introduction to lightning. Dordrecht: Springer Netherlands; 2015.

[14] Elsom DM (2001) Deaths and injuries caused by lightning in the United Kingdom: Analyses of two databases. Atmospheric Research 56 (1–4): 325–334. 10.1016/S0169-8095(00)00083-1

[15] Price TG, Cooper MA (2013) 1906 CHAPTER 142 Electrical and Lightning Injuries.

[16] DIN EN 1149–5:2018–11 (2018) Protective clothing—Electrostatic properties—Part 5: Material performance and design requirements. 10.31030/2826749

[17] Kitagawa N (1985) The nature of lightning discharges on human bodies and the basis for safety and protection. Conference Proceedings of the 18th ICLP VDE-Verlag, Berlin, 1985: 435–438. Available from: https://ci.nii.ac.jp/naid/10025827965/en/.

[18] IEC 62305:2013 (2013) Protection against lightning.

[19] Tidswell AT, Bagshaw AP, Holder DS, Yerworth RJ, Eadie L, S Murray et al (2003) A comparison of headnet electrode arrays for electrical impedance tomography of the human head. Physiological measurement. 24 (2): 527–544. 10.1088/0967-3334/24/2/363 12812436

[20] Sperandio M, Guermandi M, Guerrieri R (2012) A four-shell diffusion phantom of the head for electrical impedance tomography. IEEE transactions on bio-medical engineering 59 (2): 383–389. 10.1109/TBME.2011.2173197 22027364

[21] Hunold A, Güllmar D, Haueisen J (2019) CT Dataset of a Human Head. 10.5281/zenodo.3374839

[22] Gabriel S, Lau RW, Gabriel C (1996) The dielectric properties of biological tissues: II. Measurements in the frequency range 10 Hz to 20 GHz. Phys. Med. Biol. 41 (11): 2251–2269. 10.1088/0031-9155/41/11/002 8938025

[23] Yamamoto T, Yamamoto Y (1976) Electrical properties of the epidermal stratum corneum. Medical and biological engineering 14 (2): 151–158. 10.1007/BF02478741 940370

[24] Birgersson U, Birgersson E, Aberg P, Nicander I, Ollmar S (2011) Non-invasive bioimpedance of intact skin: mathematical modeling and experiments. Physiological measurement 32 (1): 1–18. 10.1088/0967-3334/32/1/001 21098911

[25] IT'IS Foundation (2019) IT´IS Database for dielectric properties. Available from: https://itis.swiss/virtual-population/tissue-properties/database/; checked 23.08.2019

[26] IEC 61000-4-5:2014 (2014) Electromagnetic compatibility (EMC)—Part 4–5: Testing and measurement techniques—Surge immunity test.

[27] Kuffel E, Kuffel J, Zaengl WS High voltage engineering Fundamentals. Oxford: Newnes; 2000.

[28] Machts R, Hunold A, Leu C, Haueisen J, Rock M (2016) Development of a head-phantom and measurement setup for lightning effects. IEEE Engineering in Medicine and Biology Society. 2016: 3590–3593. 10.1109/EMBC.2016.7591504 28269072

[29] Küchler A. High voltage engineering Fundamentals—Technology—Applications. Berlin, Heidelberg: Springer Vieweg; 2018.

[30] Kind D, Kurrat M, Kopp TH (2016) Voltage-time characteristics of air gaps and insulation coordination—Survey of 100 years research. 2016 33rd International Conference on Lightning Protection (ICLP). pp. 1–8. 10.1109/ICLP.2016.7791358

[31] Bazelian ĖM, Raizer JP (1998) Spark discharge. CRC Press Taylor & Francis Group LLC. Boca Raton FL USA. (6.11.2 A creeping leader, 6.11.3 A leader along a conducting surface pp. 256–260)

[32] Bhatt DL, Gaylor DC, C. Lee R (1990) Rhabdomyolysis due to pulsed electric fields. Plastic and reconstructive surgery. 10.1097/00006534-199007000-00001 2359775

[33] Bier M, Chen W, Bodnar E, Lee RC (2005) Biophysical injury mechanisms associated with lightning injury. NeuroRehabilitation 20 (1): 53–62. Available from: http://myweb.ecu.edu/bierm/papers/nre265.pdf. 15798357

[34] Abramov GS, Bier M, Capelli-Schellpfeffer M, Lee RC (1996) Alteration in sensory nerve function following electrical shock. Burns 22 (8): 602–606. 10.1016/s0305-4179(96)00055-1 8982537

[35] Lee RC (1997) Injury by electrical forces: pathophysiology, manifestations, and therapy. Current problems in surgery 34 (9): 677–764. 10.1016/S0011-3840(97)80007-X 9365421

[36] Haueisen J, Tuch DS, Ramon C, Schimpf PH, Wedeen VJ, George JS et al (2002) The influence of brain tissue anisotropy on human EEG and MEG. NeuroImage 15 (1): 159–166. 10.1006/nimg.2001.0962 11771984

